# Parasites in algae mass culture

**DOI:** 10.3389/fmicb.2014.00278

**Published:** 2014-06-06

**Authors:** Laura T. Carney, Todd W. Lane

**Affiliations:** ^1^Heliae Development, LLCGilbert, AZ, USA; ^2^Systems Biology Department, Sandia National LaboratoriesLivermore, CA, USA

**Keywords:** algae mass culture, algae parasites, parasite detection, contamination control, algal biofuels

## Abstract

Parasites are now known to be ubiquitous across biological systems and can play an important role in modulating algal populations. However, there is a lack of extensive information on their role in artificial ecosystems such as algal production ponds and photobioreactors. Parasites have been implicated in the demise of algal blooms. Because individual mass culture systems often tend to be unialgal and a select few algal species are in wide scale application, there is an increased potential for parasites to have a devastating effect on commercial scale monoculture. As commercial algal production continues to expand with a widening variety of applications, including biofuel, food and pharmaceuticals, the parasites associated with algae will become of greater interest and potential economic impact. A number of important algal parasites have been identified in algal mass culture systems in the last few years and this number is sure to grow as the number of commercial algae ventures increases. Here, we review the research that has identified and characterized parasites infecting mass cultivated algae, the techniques being proposed and or developed to control them, and the potential impact of parasites on the future of the algal biomass industry.

## Introduction

Biological constraints on mass algae production in the form of grazers, pathogens and parasites are numerous (Table 1). Parasites have been recognized as important drivers of algae population regulation in nature. For example, populations of bloom-forming algae are often associated with parasites that, along with grazers and pathogenic bacteria, play an important role in the eventual demise of the bloom (e.g., Grami et al., 2011). Similar to high density algae blooms in nature, intensive algal production is likely to be associated with higher instances of disease outbreaks caused by pathogens and parasites, as seen with seaweed cultivation in Asia (Gachon et al., 2010). In fact, fungal contamination by chytrids has been recognized as one of the most serious hurdles for producing astaxanthin from the green algae *Haematococcus pluvialis* (Han et al., 2013). Undoubtedly, the wide array of known and yet to be characterized parasites associated with algae will pose a significant biological, and thus, economic challenge to the commercial cultivation of algae in industrial settings.

**Table 1:** **Parasites reported for microalgae, including common group names and phyla, and the type of system the relationship was reported for**.

**Parasite**				
**Group/Taxonomy**	**Species**	**Microalgal host**	**System**	**Citation**
Amoebae/*Endomyxa*	*Leptophyrs vorax*	*Closterium* sp.	Laboratory culture	Hess et al., 2012
Amoebae/*Endomyxa*	*Vampyrella* sp.	Various	Natural systems	Hess et al., 2012
Aphelid/*Cryptomycota*	*Amoeboaphelidium protococcarum*	*Scenedesmus* sp.	Open raceways for mass cultivation	Letcher et al., 2013
Aphelid/*Cryptomycota*	*Amoeboaphelidium protococcarum*	*Scenedesmus* sp.	Laboratory culture	Gromov and Mamkaeva, 1970
Chytrid/*Blastocladiomycota*	*Paraphysoderma sedebokerensis*	*Haematococcus pluvialis*	Laboratory culture	Hoffman et al., 2008; Gutman et al., 2009
Chytrid/*Chytridiomycota*	*Rhizophydium algavorum*	Various	Laboratory culture	Gromov et al., 1999
Chytrid/*Chytridiomycota*	*Chytriomyces* sp. and *Zygorhizidium* sp.	Various diatoms	Laboratory culture and natural systems	Canter and Jaworski, 1979; Beakes et al., 1988; Bruning, 1991; Grami et al., 2011; Kagami et al., 2011
Chytrid/*Chytridiomycota*	*Entoplyctis apiculata*	*Chlamydomonas* sp.	Natural system	Shin et al., 2001
Chytrid/*Chytridiomycota*	*Phlyctidium scenedesmi*	*Scenedesmus* sp.	Open raceways for mass cultivation	Fott, 1967; Ilkov, 1975
Chytrid/*Chytridiomycota*	*Rhizophidium* sp.	*Scenedesmaceae*	Closed photobioreactors for mass cultivation	Carney et al., 2014
Labyrinthulid/ *Labyrinthulomycota*	*Labyrinthula* Cienk.	*Cyanobacteria*	Natural system	Raghukumar, 1987
Oomycete/*Oomycota*	*Ectrogella* sp.	*Pseudo-nitzschia*	Natural system	Hanic et al., 2009
Oomycete/*Oomycota*	*Lagenisma coscinodisci*	*Coscinodiscus centralis*	Natural system	Gotelli, 1971
Sindinids/*Alveolata*	*Amoebophrya* sp.	*Dinoflagellate*	Natural system	Guillou et al., 2008; Chambouvet et al., 2011
Sindinids/*Alveolata*	*Amoebophrya ceratii*	*Dinoflagellate*	Natural system	Chambouvet et al., 2008

## Review of some known algae parasites

### Fungi

In freshwater environments, zoosporic fungi (Chytridomycota) and fungi-like organisms (including oomycetes, labyrinthulids, thraustochytrids and phagomyxids) are well known to parasitize microalgae. However, in marine systems the vast majority of important predators, pathogens and parasites have not been well characterized. In fact, it is estimated that only 0.6% of fungi studied are marine and these are only distantly related to known and cultured fungi (Richards et al., 2012). This suggests that there is an enormous amount of undiscovered marine microbial diversity. This is significant because the majority of algal species being utilized for biofuel production are marine in origin, posing severe monetary risk for the algal industry, which will have to contend with enemies it is not prepared for.

Members of the Chytridiomycota are extremely common fungal parasites in freshwater systems that prey on algae (Table 1). Their host ranges can be narrow (e.g., *Paraphysoderma sedebokerensis;* Hoffman et al., 2008; Gutman et al., 2009) or wide (e.g., *Rhizophydium algavorum*; Gromov et al., 1999) depending on the species. Chytrids produce motile dispersing life stages know as zoospores, are either saprotrophic or parasitic and are important contributors to aquatic food chains and carbon cycling (reviewed by Gleason et al., 2008). Despite high infection rates in natural algae populations, it is unclear how severe the effect of parasitic chytrids is (e.g., Kagami et al., 2011), however their impact may be amplified in commercial settings. For example, the chytrid *Phlyctidium scenedesmi* has been noted to cause severe production loss of *Scenedesmus* in open pond systems (Fott, 1967; Ilkov, 1975). Microbiome analyses and chitin staining recently detected several chytrids that co-occurred with loss of productivity of mixed green algae (family: *Scenedesmaceae*) growing in a prototype Offshore Membrane Enclosures for Growing Algae (OMEGA) system (Carney et al., 2014). These included the parasitic chytrid *Rhizophidium* sp. and a saprotrophic cytrid that was either *Powellomyces* or *Entophlyctis* sp. Unfortunately, the thick-walled cysts of chytrids can withstand many disinfection techniques (Fott, 1967), and once established may become a persistent problem for subsequent cultures.

Aphelids are a sister taxon of the chytrids in the Cryptomycota and are known as intracellular parasites that feed on microalgae (Karpov et al., 2013) (Table 1). A novel aphelid, *Amoeboaphelidium protococcarum*, was recently discovered and described infecting *Scenedesmus dimorphus* in commercial ponds in New Mexico, USA (Letcher et al., 2013). The proliferation of *A. protococcarum* in the ponds was correlated with a decrease in algae. This finding highlights that the need to identify new parasites will likely increase with the growth of the commercial algae industry.

Labyrinthulids are members of the fungal class *Labyrinthu-lomycetes*, commonly known as the slime molds, and include some important parasites of marine algae and plants (Table 1). Members of the genus Labyrinthula cause wasting disease in seagrasses and parasitize green algae and cyanobacteria (Raghukumar, 1986, 1987). These fungi have not yet been described in commercial systems, but closely related saprotrophic Thraustochytrids have been detected in *Nannochloropsis* sp. raceways using microbiome analysis (Carney et al., in preparation).

Oomycetes are a group of fungus-like organisms (technically in Kingdom Chromista) commonly referred to as water molds that include many known parasites of a wide variety of prey, including land plants and marine seaweeds and microalgae (Gachon et al., 2009; Hanic et al., 2009; Li et al., 2010) (Table 1). Oomycetes have caused losses to the seaweed industry that range from 10 to 60% annually for some countries (Gachon et al., 2010). Reported infection frequencies by the oomycetes *Ectrogella* and *Lagenisma* in natural populations of marine diatoms have ranged from <1 to 99% (Hanic et al., 2009, reviewed by Li et al., 2010). Like Labyrinthulids, Oomycetes have not yet been described in algae cultivation systems but their presence will likely be discovered as the commercial microalgae industry grows and molecular detection techniques are more frequently applied.

### Amoebea

Vampyrellids are naked filose amoebae that perforate algae cell walls with spike-like pseudopodia in order to extract cellular content (Hess et al., 2012), hence the graphic name assigned to this group. Vampyrellids are common in freshwater and some are thought to be marine (Table 1). Although this group is not very well understood, Vampyrellids may become more notorious as the commercial algae industry grows.

### Other parasites

The *Syndiniales* are alveolates, closely related to dinoflagellates, known to infect bloom-forming dinoflagellates in nature, exerting population regulation in only a few days by causing cell death without reproduction (reviewed by Gachon et al., 2010; Miller et al., 2012). *Amoebophrya* sp. were recently discovered to be able to survive in dormant cysts of their hosts for many months, causing immediate reinfection cycles as soon as the cysts emerged (Chambouvet et al., 2011), suggesting the infection by these parasites could be perpetuated very easily. *Amoebophrya ceratii* was recently discovered as the agent preventing seasonal algae blooms by an invasive dinoflagellate in an estuary in France (Chambouvet et al., 2008). However, the parasite was also found to infect every other dinoflagellate species in the area, including many native species. The wide host range and recurrence of infection of the sindinids pose many challenges to commercial production of dinoflagellates.

## Detection of algal parasites

### Microscopy and staining

Calcofluor white is commonly used to visualize chytrids by staining the chitin in the cell walls of certain life stages (Kagami et al., 2004; Rasconi et al., 2009). Gerphagnon et al. (2013) proposed a double staining method to assess chytrid infection rates of cyanobacteria using Calcofluor white and SYTOX green, a nucleic acid stain. The authors used a combination of UV and blue light to show chytrid zoospores (green) inside sporangia (blue). However, for some algae Calcofluor white is problematic when cellulose is the primary cell wall component, such as for *Haematococcus pluvialis*, because cellulose can be stained as well as chitin and may obscure detection (Damiani et al., 2006). In addition, Calcofluor white cannot stain fungi lacking chitin. As an alternative, staining chytrid sporangia with nile red, even at very young stages, can be used as an early detection method for algae (Gutman et al., 2009). Congo red staining has been used to visualize oomycetes parasitizing seaweeds (Gachon et al., 2010) and may be useful for parasites of microalgae.

### Flow cytometry

Sieracki et al. (1998) have developed an automated flow cytometry and microscopy system known as FlowCAM (Fluid Imaging Technologies) for the enumeration and characterization of suspended particles, usually phytoplankton, in the 20–200 μM size range. The FlowCAM operates by flowing samples through a 3 × 3 mm glass chamber illuminated by a green laser and where relative fluorescence data, dimensions and a digital microscopic image of each particle are captured. Image analysis software, included with the instrument, carries out a pixel-based comparison correlation between captured images and a previously collected reference image set resulting in a percent similarity score. Images, that exceed a user determined similarity threshold, are considered to be a match for the target organism and can be displayed for visual confirmation by the user. In this manner FlowCAM systems can be used for semiautonomous identification and enumeration of target species. FlowCAM systems have been used in a variety of applications in marine and aquatic sciences including the detection and enumeration of the toxic dinoflagellate *Karenia brevis* in laboratory cultures, spiked natural phytoplankton assemblages and field samples from the Gulf of Mexico (Buskey and Hyatt, 2006). More recently FlowCAM analysis has been applied to the monitoring of algal mass culture systems for the early detection of algal predators (Day et al., 2012). A different flow-through microscopy system has been developed and demonstrated for the automated monitoring of cell count, size and morphology in microalgal culture (Havlik et al., 2013) To date there are no reports in the literature of the application of FlowCAM technology to the detection of parasites in algal mass culture but the technology may be applicable to visually distinctive species.

### Molecular–based detection and monitoring

Modern molecular methods that have been developed for ecological studies can offer alternatives to optical based detection systems for the identification and detection of parasites in algal mass culture. Initial molecular identification can be carried out by Sanger sequencing of isolated DNA templates or by shotgun approaches based on next generation sequencing technologies. Once identified and characterized, systems that utilize amplification of or oligonucleotide hybridization to specific target regions can be employed for the detection of parasitic species. There are a variety of target regions that have been developed for either identification or detection; most of which utilize the ribosomal RNA (rRNA) encoding region. The three main targets in this region are the small subunit (SSU) rRNA large subunit (LSU) rRNA genes and the internal transcribed spacer (ITS).

In general, the SSU rRNA gene contains nine hypervariable regions (V1–9). However, unlike the prokaryotic SSU rRNA, the eukaryotic form lacks the V6 region so regions V4 and V9 are the most common individual hypervariable regions used for the phylogenetic analysis of eukaryotes (Amaral-Zettler et al., 2009; Stoeck et al., 2010; Pawlowski et al., 2011; Orsi et al., 2013). The V4 is the longest of the hypervarible regions, displays the highest degree of length variation and sequence heterogeneity (Nickrent and Sargent, 1991) and is generally sufficient for the genus level identification of an organism (see examples in Carney et al., 2014). The shorter V9 region is sometimes used in combination with V4 but, since it lies at the extreme 3′ terminus of the SSU rRNA gene, it is often not included in less than full-length amplicons used for sequencing. Thus, data is often missing for the V9 region in nucleotide sequence database entries. Two variable regions (D1 and D2) in the LSU rRNA gene are also used for phylogenetic analysis (Ludwig and Schleifer, 1994; Sonnenberg et al., 2007; Putignani et al., 2008). A number of tools have been developed to assist in rRNA based phylogenetic analysis. Perhaps the most heavily utilized of these is the SILVA database, (http://www.arb-silva.de/), a curated and downloadable repository of SSU and LSU rRNA gene sequences (Quast et al., 2013). The SILVA website also includes webtools for the *in silico* testing of primer and probe sequences for specificity. In adddition, probeCheck, http://131.130.66.200/cgi-bin/probecheck/content.pl?id=home (Loy et al., 2007) can also be used in a similar fashion to aid in the design of SSU and LSU rRNA probes.

For finer levels of phylogenetic discrimination, the ITS region is commonly utilized. This region consists of two hypervariable spacers, ITS1and ITS2, 5′, and 3′ of the gene encoding the 5.8 s ribosomal subunit. Currently there are three online databases of ITS sequences: UNITE (http://unite.ut.ee/) contains primarily fungal ITS regions (Abarenkov et al., 2010), the ITS2 database (http://its2.bioapps.biozentrum.uni-wuerzburg.de/; Koetschan et al., 2012), and ITSoneDB, (http://itsonedb.ba.itb.cnr.it/) focuses on the ITS1 region.

A full-length amplicon including both ITS1 and ITS2 regions and the 5.8 s subunit is approximately 650 bp in length, which is beyond the current read length limits of many of the next generation sequencers. Consequently, individual ITS regions have been analyzed by next generation sequencing (Lindner et al., 2013). Reports indicate that community analyses based on ITS1 vs. ITS2 yield different taxonomic compositions from each other as well as from those based on the full length ITS region.

Because of its potential for high sensitivity, quantitative PCR (qPCR) is a powerful and widely employed detection method (for review of qPRC detection of waterborne agents see Botes et al., 2013). Careful sample preparation methods including primer design and validation and selection and testing of reagents are required in order to limit background contamination and achieve the highest levels of sensitivity. qPCR reactions can be multiplexed for the simultaneous detection of multiple species within a single reaction. qPCR has been used in commercial algae production to quantify changing densities of algae and their parasites in mass-culture, for example, *Amoeboaphelidium protococcarum* (Letcher et al., 2013) and chytrids (Shurin et al., 2013). qPCR could be a very important detection tool when the parasite is known. Alternative detection methods include hybridization-based systems, such as the phylochips, which utilize arrayed oligonucleotide probes (Loy et al., 2002; Metfies et al., 2007). Although, without an integrated amplification step, these methods are not as sensitive as qPCR; they are however designed to be highly multiplexed for the simultaneous detection of a diversity of species and thus could be both cost and time effective, often required in commercial settings.

## Solutions to parasite contamination

### Salvage harvest

Perhaps the most obvious, least technologically demanding and least satisfying response to parasite contamination is salvage harvest. This is, of course, to simply harvest the algal biomass upon detection of a parasitic species and prior to serious loss of biomass. Successful salvage harvest is dependent on both the early detection and quantification of the contaminating parasite and the operator experience necessary to determine optimal harvest time by balancing the maximizing of biomass yield against the potential for catastrophic loss. Although this method reduces the impact of an infection, it still results in removal of a mass culture system from active production. The system must be disinfected prior to return to production or used to produce an alternative, non-susceptible species. Disinfection of unlined, open pond systems can be challenging and techniques may be limited to drying and exposure to solar radiation.

### Chemical agents

Natural defenses of algae to parasite infections include abscisic acid (ABA) production during some life cycle stages (Pouneva, 2006). ABA may also have protective effects when applied exogenously. An anti-fungal protein isolated from a marine bacterium has been used to protect commercially grown red seaweed from the oomycete causing red rot disease (Woo and Kamei, 2003). The addition of copper sulfate to growth media has been reported to both stimulate algae productivity and serve as a fungicide against chytrids (Fott, 1967). An unspecified chemical fungicide was successful at controlling chytrid densities in commercial ponds of *Scenedesmus* sp. (Shurin et al., 2013). The surfactant Triton-N was proposed for treating algae inoculum before scale-up when it is still a small volume in order to reduce chytrid productivity (Benderliev et al., 1993). Several commercially available disinfectants were found to control the spread of the chytrid infecting amphibians worldwide, *Batrachochytrium dendrobatidis* (Webb et al., 2012) and may have application to algal mass culture.

Ozone treatment has been employed for the destruction of invasive species in ballast water but the application of the method to algal parasites has not been reported (Tsolaki and Diamadopoulos, 2010). Germicidal ultra violet radiation subtype C (UVC; 280–100 nm wavelength) has also been proposed for the control of invasive species but has not been demonstrated for use against algal parasites (Liebich et al., 2012). Successful employment of UVC in contaminated mass algal cultures would depend on greater resistance to irradiation of the alga vs. the parasite. This appears to be possible because of the greater level of pigmentation in microalgae vs. many parasite species. Alternatively UVC could be utilized to eliminate sources of contamination in liquid nutrient stocks or source water for cultivation.

### Physical methods

Physical disruption (sonication) has been studied for the destruction of invasive and deleterious organisms in ballast water (Holm et al., 2008). Dose is dependent on cell size with larger organisms requiring less energy input for disruption than those that are smaller. This makes sonication particularly attractive for the control of predators but potentially less so for the parasites, which may not display as great a size differential. Physical removal methods such as screening have also been demonstrated for the removal of large biovolume predators such as rotifers but, again, this dependence on size differential may limit its utility in treating parasite infections.

### Biological control

#### Selective breeding/modification

The utilization of selective breeding, or other methods for the genetic manipulation of microalgae, to develop resistance to parasite infection in algal mass culture has yet to be reported. Microalgae have several characteristics that could potentially lend themselves to such an approach (for review see Larkum et al., 2012). Generally speaking, generation times are relatively short and UV or chemical mutagenesis methods have been developed in a number of species (Huesemann et al., 2009). In a handful of strains, including *Chlamydomonas reinhardtii, Phaeodactylum tricornutum, Thalassiosira psuedonana, Nanochloropsis salina*, and *Dunaleiella* sp., transformation methods have been developed enabling the genetic engineering of these species (for review see Qin et al., 2012). The success of any genetic manipulation technique is dependent on the ability to select for resistance; such selection would require that the target parasite species is maintained in culture. Other limitations to genetic modification approaches include the potential for tradeoffs; a mutant microalgal strain that demonstrates good parasite resistance may not perform well in other aspects. The breadth of resistance could be quite narrow with resistance to one parasite species extending only to closely related species. Finally, because of selective pressure, there is the potential for the parasite to rapidly evolve to overcome the host algae resistance.

#### Biological agents

Bio-control is widely used in agriculture and on public lands to manage unwanted pests ranging from invasive plants to mammals. Bio-control of plant pathogens has been gaining momentum (36% of existing bio-control agents were developed only in the last 5 years) and is often preferred over chemical means of control. Residues from chemical control methods may hinder downstream product processing, regulation of these chemicals has become increasingly restrictive, and target pests are likely to develop chemical resistance (Fravel, 2005). Alternatively, organisms that prey on and parasitize microalgae have their own set of predators and parasites that may be used to control them in commercial settings.

Zooplankton may be used as an effective control of algae parasites as they prey on fungal spores, particularly chytrid zoospores (Kagami et al., 2004), which have been recognized as important resources in natural food chains (Gleason et al., 2008). Due to its efficient grazing of chytrid zoospores, *Daphnia* has been tested in mesocosms as a potential biocontrol agent of *Batrachochytrium dendrobatidis* (Hamilton et al., 2012). However, in this example, Daphnia also served as prey for developing tadpoles (Hamilton et al., 2012). Care must be taken in applying zooplankton to control fungus in algae cultures as many of these also rapidly ingest algae and can have equally, if not more, damaging effects on algae biomass than chytrid infections do.

Hyperparasites are organisms that parasitize other parasites, although these have not been studied very thoroughly. Work is needed to describe algae-parasite-hyperparasite relationships that may be common to commercial algae production ponds and that show promise as a solution paradigm to fungal infections of algae and from which future bio-control tools can be developed for additional algal predators. Evidence suggests hyperparasite infections do not kill their fungal host but instead reduce their reproductive success by efficiently co-opting the cytoplasm of infected cells and thereby indirectly reducing infection rates of algae (Gleason et al., 2012). Examples of such relationships are common in freshwater environments and a marine example has been described (Kagami et al., 2007; Gleason et al., 2012). In addition, metagenomic evidence suggests hyperparasites are common in marine communities (James and Berbee, 2011). The host range of hyperparasites has been determined to be narrow with each species of hyperparasite infecting only closely related species. Some hyperparasites produce resting spores that can withstand desiccation and may potentially be used to inoculate cultures of infected algae in order to control the fungal parasite. In fact, fungal species that have resting spores as part of their life cycles are noted as being easy to formulate as bio-control agents because they are easier to ship and have lower risk of contamination by bacteria and other fungus (Fravel, 2005). As our interest, and perhaps future dependence, on biofuels grows, the complex algal pond relationships will need to be understood and controlled in order to attain sufficient algal productivity.

Allelopathy is the production of one or more biochemicals by an organism that affects, either positively or negatively, the survival, growth or reproduction of another species. Negative allelopathy has been proposed as a potential method of controlling deleterious species in algal mass culture systems (Mendes and Vermelho, 2013). However, alleopathic relationships between microalgae and parasites have not been reported and thus allelopathy is only a hypothetical mechanism for the control of parasites in algal mass culture.

## Conclusions

Here we have reviewed some known parasites of microalgae, as well as some taxonomic groups that will likely become better known (i.e., infamous) as commercial production of microalgae increases worldwide. Members of the fungi, including chytrids and oomycetes, appear to be the most common and potentially, least controllable, group of parasites. Early detection of parasites is essential to ensure the efficacy of possible treatments of the infection. Traditional detection methods such as microscopy and staining can be used to visualize algal parasites, however this technique may be too labor intensive to perform on a routine basis for most commercial operations. For routine detection, more automated systems would be ideal (i.e., flow cytometry). If the budget allows, molecular-based techniques are the most informative and sensitive for the purposes of identifying which parasites may be present using Sanger, shotgun or next generation sequencing and then monitoring for these specifically using qPCR or phylochip technology.

The economic feasibility of the various parasite detection and control methods is largely driven by two factors; the volume of the algal mass culture system and value of the final product or products. Algal biofuels is a prime example of a low value product that must be produced at large scale. To compete with gasoline at $0.53US per liter it will be necessary to produce dry algal biomass at $0.14US per kilogram (Sun et al., 2011). Given these limitations, it is widely, but not universally, held that large-scale open ponds may be the only economically feasible means of production. Because of this combination of the large scale of cultivation, the relatively low productivities that can be achieved in open systems (as opposed to closed PBRs), and the low value of the final product, the options for parasite control in biofuels applications are economically constrained. In addition, the likelihood of infection is the greatest in open systems. In such an application, intervention strategies must be targeted and inexpensive. Conversely, higher value products such as nutraceuticals, may accommodate a larger range of parasite control strategies while remaining economically viable. These operations, which tend to feature lower volume cultivation units, can take advantage of broadly applied prophylactic methods such as filtration, UV and chemical pretreatment of the source water.

In terms of low cost countermeasures, salvage harvest may, of course, be the least expensive but least satisfactory method of intervention. The obvious limitations of this practice continue to fuel the drive to seek alternative crop protection strategies. In the near-term, intervention with various biocidal chemicals is likely to be the most effective alternative. This is largely driven by the fact that several such chemicals already exist and are utilized in terrestrial agriculture. However, if the final products or co-products are intended for human or animal consumption, this may limit the application of chemical countermeasures. In addition, it may be economically infeasible to routinely treat all cultivation units and such extensive use of chemicals could result in the development of resistant parasite strains.

It is likely that the best long-term strategy for control of parasites in production facilities will take the form of an integrated pest management strategy. Such a strategy would include the cultivation of resistant strains, the limited use of chemical agents, the development of biological control systems and crop rotation to limit the accumulation of parasites or the development of resistance to countermeasures. By combining different management strategies, shortcomings of any one strategy may be overcome and reliance on chemicals can be reduced (reviewed by Chandler et al., 2011). Resistant algal strains could be developed either by classical mutagenesis and selection strategies or by genetic engineering techniques. The former would not be considered a genetically modified organism (GMO) for regulatory purposes.

Routine monitoring and early detection of pest species is a clear requirement for large-scale cultivation. Knowledge gained from long-term operations will allow for the identification of common pest species and the environmental conditions in which they are most prevalent. To some degree this gives the operators predictive capability. In that manner only the cultivation units that require it are treated. Daily microscopic analysis is a standard practice at many production facilities. However, this process is labor intensive and requires a certain degree of expertise. Alternative parasite detection method based molecular assays such as PCR will likely find application in the production of high value products but do require significant capital outlay and may be too expensive for large-scale operations. The same may be said of image recognition systems with the additional caveat that they may be unable to identify morphologically indistinct species. Clearly there is an unmet need in the nascent algal production industry for a low cost user-friendly “dipstick” assay for the major parasite species. In addition, more work is needed in developing treatment protocols that target specific host-parasite relationships, as both the parasite and some algal hosts may be affected negatively by the treatment.

Despite a paucity of publically available data on the economic impact of parasitism on the nascent algae biomass industry, the consensus is that biocontaminants, in general, constitute an economic barrier to commercialization (ANL et al., 2012; Gao et al., 2012). Some insight into the potential magnitude of the financial impact may be gained from the *Porphyra* (nori) industry in asia which loses 10%, on average, of its annual production to parasitism by oomycetes, with losses up to 64% in certain regions during some years (reviewed by Gachon et al., 2010). Commercial algal mass culture operations would clearly benefit from a more complete understanding of algal parasites including regional, environmental and seasonal variation in occurrence of parasite infestations and a characterization of the susceptibility of common production strains to different parasites. Such an understanding would require a systematic approach to the analysis and characterization of pond infections and a certain amount of data sharing among pond operators. At minimum, a shared database of molecular probes and PCR primer sequences for detection of parasites would be beneficial. Perhaps as the algal mass culture industry becomes more economically feasible and therefore more important there will be greater impetus to take such an approach to the problem.

### Conflict of interest statement

The authors declare that the research was conducted in the absence of any commercial or financial relationships that could be construed as a potential conflict of interest.
